# Can Enzymatic Modified Pectin in Mediterranean Fruits Be Therapeutic Against Stomach Cancer?

**DOI:** 10.1002/fsn3.4507

**Published:** 2024-10-14

**Authors:** Selime Üstün, Hamit Emre Kizil, Enes Dertli

**Affiliations:** ^1^ Department of Food Engineering Bayburt University Bayburt Türkiye; ^2^ Vocational School of Health Services Bayburt University Bayburt Türkiye; ^3^ Department of Food Engineering Yıldız Technical University İstanbul Türkiye

**Keywords:** antioxidant, cytotoxicity, heterosaccharides, pectin, stomach cancer

## Abstract

The aim of this study was to evaluate the cytotoxic effects of enzymatically modified pectin products derived from grapefruit, jujube, and kumquat on the MKN‐45 gastric cancer cell line in vitro. FTIR analysis revealed that the spectra of the pectins produced were comparable to those of commercial pectin and included the characteristic peaks identified in the literature. The galacturonic acid content was measured as 612 mg/g in grapefruit pectin, 544 mg/g in jujube pectin, and 704 mg/g in kumquat pectin. DSC analysis indicated that all pectin samples and their modifications exhibited one endothermic peak and one exothermic peak. Cytotoxicity assessments revealed that the enzymatically modified grapefruit pectin demonstrated significant cytotoxic effects across all tested concentrations, with 0.075 mg/mL being the most effective. For kumquat pectin, all tested concentrations showed cytotoxic properties, with 0.3, 0.15, and 0.075 mg/mL being the most effective. In the case of jujube pectin, cytotoxic effects were observed at all concentrations except for 1.2 mg/mL.

## Graphical abstract

In vitro cytotoxic effects of enzymatically modified pectin products from grapefruit, jujube and kumquat on MKN‐45 gastric cancer cell line at different concentrations were determined.

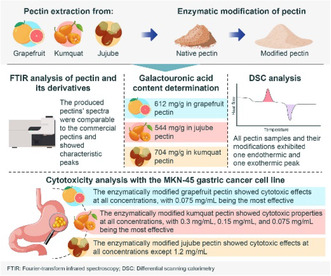

## 1 Introduction

Evaluation of agricultural industry residues is a current issue, especially in developing and developing countries. Large amounts of food residues are formed as a by‐product along the food production line. A significant part of these residues, which sometimes cause environmental pollution, are destroyed immediately or used to produce products such as animal feed and fertilizer with low economic value. Evaluation of these residues may be important in terms of preventing environmental pollution as well as producing and diversifying products with high added value (Cin and Gezer 2017).

An example of one of these products is pectin. Pectin is a heteropolysaccharide that naturally occurs in plant cells, plays a significant role in the growth and development of plant tissues, provides mechanical strength to the plant, and is located in the cell wall and intercellular regions (Bagherian et al. 2011; Zhang et al. 2013). The pectin molecule is one of the most important components of the plant cell wall and typically forms during the initial stages of primary cell wall development, constituting one‐third of the dry matter in the cell wall (Celestino et al. 2006). Pectin, one of the complex polysaccharides found in nature, is extracted from fruits and vegetables under suitable conditions and does not exist in free form (Lee 2012). Pectin, a plant‐based stabilizer, binds cells together. Its quantity is higher in fast‐growing plants with a high moisture content (Begum et al. 2017). Pectin, which holds an important place among cell wall polysaccharides and is found in the cell walls and intercellular regions of all young plants, is widely used today in food and biomedical applications (Shpigelman et al. 2015).

Pectin, one of the water‐soluble dietary fibers found in the structure of fruits and vegetables, is known to have many effects on human health. In addition to its effects such as lowering blood cholesterol levels and preventing the formation and spread of cancer, daily oral intake of pectin is known to significantly reduce intestinal infections in infants and children by strengthening the immune system, slow diarrhea, facilitate the excretion of toxic substances, reduce lead absorption, and have a preventive effect on stroke, coronary heart disease, and obesity (Thakur et al. 1997). It has been also determined that pectin is used as a substrate for fermentation by various bacteria in the gut flora, and that pectin supplementation reduces the number of pathogenic microorganisms in the intestines of infants and children, significantly reducing diarrhea. Pectin increases mineral absorption, shortens gastric emptying time, and also has positive effects on the digestive system (Wüstenberg 2014).

In a study conducted with pectin of different degrees of esterification obtained from lemon peels, mango peels, and sugar beets, the mineral absorption of mice fed with heavy metals (Pb and Cd) and bivalent (Zn, Cu, Ca, and Fe), and monovalent (K and Na) minerals in their diets was examined. At the end of the study, it was observed that the number of heavy metals decreased with pectin intake, pectin with a low degree of esterification was more effective in mineral absorption, and the type and dosage of pectin were important for preventing the absorption of essential dietary minerals, and that pectin could be useful in heavy metal poisoning (El‐Zoghbi and Sitohy 2001). In another study, blood values were examined by adding grapefruit pectin to the diet of experimental animals, and while the cholesterol level was found to be 168 mg/dL in the group with added pectin, it was 249 mg/dL in the group without added pectin (Baekey et al. 1988). It has been determined that modified citrus pectin consumption within a certain time interval (1–6 days) facilitated the excretion of elements such as lead, cadmium, arsenic, and mercury, which can cause toxic effects, through urine, and reduced tumor formation in rats fed with modified citrus pectin (Nangia‐Makker et al. 2002). Pectin has been found to reduce blood clotting time in hemorrhages and is effective in controlling bleeding, while pectin sulfate, on the contrary, increases clotting time and can be used as a substitute for heparin (Brake and Fennema 1993). Analyses conducted on humans and dogs have determined that there is no enzyme in saliva or gastric juice that breaks down pectin. In another study conducted on humans and animals, pectin was broken down by bacterial enzymes in the intestines, and the main products formed from the pectin molecule as a result of this bacterial fermentation were CO₂, CH₂O₂ (formic acid), and CH₃COOH (acetic acid) (Thakur et al. 1997).

Moreover, pectin has many applications in pharmacology. Daily consumption of 6 g of pectin in the diet significantly reduces blood cholesterol levels, while this effect is not observed when less than 6 g is consumed daily. Pectin is known to act as a natural prophylactic component by binding toxic cations in cases of poisoning. Pectin is also used alone or in combination with gelatin in the production of drug capsule materials (Ashford et al. 1994). In another study, it has been reported that modified pectin functions as an anti‐cancer agent by binding to the pro‐metastatic protein galectin‐3. It has also been reported that pectin is used as a transmucosal delivery system to reduce cancer pain (Videcoq et al. 2011).

Studies on the biological activities of enzymatically modified products of pectin have recently been highly focused. A 2022 study revealed that raspberry pectin extracted using enzymes had an increased presence of arabinan side chains and demonstrated enhanced anti‐inflammatory properties (Wu et al. 2022). In the same year, Zhang et al. (2022) reported that enzymatic and alkaline modification of hawthorn pectin activated macrophages. Enzymatically modified pectin, derived from orange, lemon, lime, and sugar beet pectin, also alters the structure and function of the gut microbiota and promotes the growth of *Akkermansia* and *Lactobacilli* (Larsen et al. 2019). In another study, Tan et al. (2018) found that enzymatically modified pectin molecule increased the strength of calcium ion cross‐linking network and provided prolonged drug release. Ferreira‐Lazarte et al. (2018) also suggested that enzymatic modification of artichoke pectin greatly promoted the growth of *Bifidobacterium*, *Lactobacillus*, and *Bacteroides/Prevotella*. Alkaline treatment in the modification of sugar beet pectin significantly amplified its anticancer properties by promoting cellular apoptosis. Conversely, enzymatic removal of galactose and arabinose from the pectin diminished its efficacy against cancer cells, underscoring the critical role of the RGI region—rich in neutral sugars—in maintaining the biological activity of pectin (Singh et al. 2019). Cui et al. (2020) stated that citrus pectin extracted with alkali + cellulase exhibited compact structure and good fermentation properties and suggested that the compact conformation contributes to fermentability and is effective in supporting intestinal health in this context. In another study, alkali treatment of sugar beet pectin extract markedly improved its cellular effects by inducing apoptosis, while leaving the cell cycle unaffected. This treatment elevated the ratio of RGI to HG content, highlighting the significance of neutral sugar side chains at the RGI regions in contributing to the bioactivity of the pectin (Maxwell et al. 2016).

Therefore, in this study, we aimed to obtain and characterize grapefruit, jujube, and kumquat pectin and their enzymatic modified products and to determine their in vitro cytotoxic effects on MKN‐45 gastric cancer cells.

## 2 Materials and Methods

### 2.1 Pectin Extraction

Pectin extraction was conducted following the method described by Kliemann et al. (2009) with some modifications. Fruits (grapefruit, jujube, and kumquat) were peeled and dried in an oven at 55°C overnight. The pH of distilled water was adjusted to 1.88, and the fruit peels were added at a ratio of 1:25. The extraction was performed at 87°C for 6 h using a magnetic stirrer. After cooling the extract to 20°C, it was centrifuged at 10,000 rpm for 10 min. The supernatant was transferred to a beaker and 96% ethanol was added at four times the volume of the supernatant. The precipitated pectin was filtered using a muslin cloth, placed in petri dishes, and dried in an oven at 55°C overnight. The dried pectin was then ground into a powder (Kliemann et al. 2009; Yılmaz et al. 2017).

### 2.2 Pectin Modification

Enzymatic modification of pectin was carried out based on the method by Buchholt et al. (2004), with minor adjustments. One gram of pectin was dissolved in 100 mL of distilled water, and the pH was adjusted to 4.2 using 5% NaOH. Next, 50 μL of pectinase enzyme was added, and the mixture was incubated at 45°C for 18 h on a magnetic stirrer. After the incubation, the temperature was increased to 65°C, and the pH was adjusted to 5.5 to inactivate the enzyme. After cooling the mixture to 40°C, four times the volume of ethanol was added to precipitate the pectin. The mixture was filtered using coarse filter paper, and the modified pectin was dried at 55°C and then ground into a powder (Buchholt et al. 2004).

### 2.3 Characterization of Pectin

#### 2.3.1 FT‐IR Analysis of Pectin and Its Derivatives

An ATR‐FT‐IR spectrophotometer (PerkinElmer, USA) was used to analyze the chemical groups of pectin. The results were cross‐referenced with the instrument's library and matched to pectin standards.

#### 2.3.2 Determination of Galacturonic Acid Content

The galacturonic acid content of the pectin samples was determined based on a modified method by Monsoor, Kalapathy, and Proctor (2001). Pectin (30 mg) was dissolved in 12 mL of 0.1 M NaOH and heated for 120 min. After adjusting the pH to 4.2 using 0.1 N HCl, 1 mg of pectinase and 25 mg of cellulase were added to initiate enzymatic activity. The mixture was incubated at 55°C for 25 h in a shaking incubator, wrapped in aluminum foil to maintain darkness. Post‐incubation, the sample was filtered first through coarse filter paper, then through a 0.45‐μm filter to prevent column clogging during HPLC analysis (Agilent Technologies, USA). Galacturonic acid was quantified using HPLC with a PDA detector and a C18 column (5 μm particle size). The mobile phase was ultrapure water, adjusted to pH 2.2 with 0.1 N HCl, with a flow rate of 0.6 mL/min and a wavelength of 200 nm.

#### 2.3.3 Differential Scanning Calorimetry (DSC)

DSC analysis was performed to assess the thermal properties of the extracted and modified pectins (PerkinElmer, USA). The temperature was increased from 30°C to 400°C at a heating rate of 5°C/min, using aluminum as the reference material under a nitrogen atmosphere (Sharma and Ahuja 2011).

### 2.4 Cell Culture and Viability Analysis

The MKN‐45 gastric cancer cell line used for cell culture studies was obtained from Yeditepe University, Department of Genetics and Bioengineering Laboratory. Cells were grown in RPMI 1640 medium (Gibco) supplemented with 10% FBS (Gibco) in 25 mL flasks. Trypsin–EDTA solution (Gibco) was used for cell detachment following a PBS (Sigma) wash. The flasks were incubated at 37°C in a 5% CO_2_ environment for 24–48 h. Cell viability was assessed using trypan blue (Gibco) before each passage. After centrifugation, cells were stained with trypan blue and counted using a hemocytometer (Mani et al. 2021; Maric et al. 2020).

Cell viability was assessed using the CVDK‐8 kit, which utilizes the WST‐8 method, obtained from Ecotech Biotechnology (Erzurum, Türkiye). Initially, the H‐460 cell line was seeded into 96‐well plates and incubated for 24 h to allow for proper cell adherence. Subsequently, synthesized molecules, dissolved in DMSO (Sigma), were prepared at five different concentrations (200, 100, 50, 25, and 12.5 μg/mL) and added to the wells. The plates were then incubated for an additional 24 h. After 72 h, cell viability was determined according to the kit's protocol. Specifically, 10 μL of CVDK‐8 reagent (Ecotech Biotechnology) was added to each well containing cells exposed to the synthesized molecules. The cells were then incubated for a further 3 h. Following incubation, the absorbance of each sample was measured at 450 nm using a spectrophotometer (Thermo Fisher Multiskan Go, USA) to evaluate cell viability (Barlak et al. 2021).

### 2.5 Statistical Analysis

Cytotoxicity experiments were performed in triplicate, and the data are presented as mean ± standard deviation. Statistical analysis was conducted using GraphPad Prism software, with Student's *t*‐test. A *p* value ≤ 0.05 was considered statistically significant.

## 3 Results and Discussion

### 3.1 FT‐IR Analysis of Pectins

**FIGURE 1:**
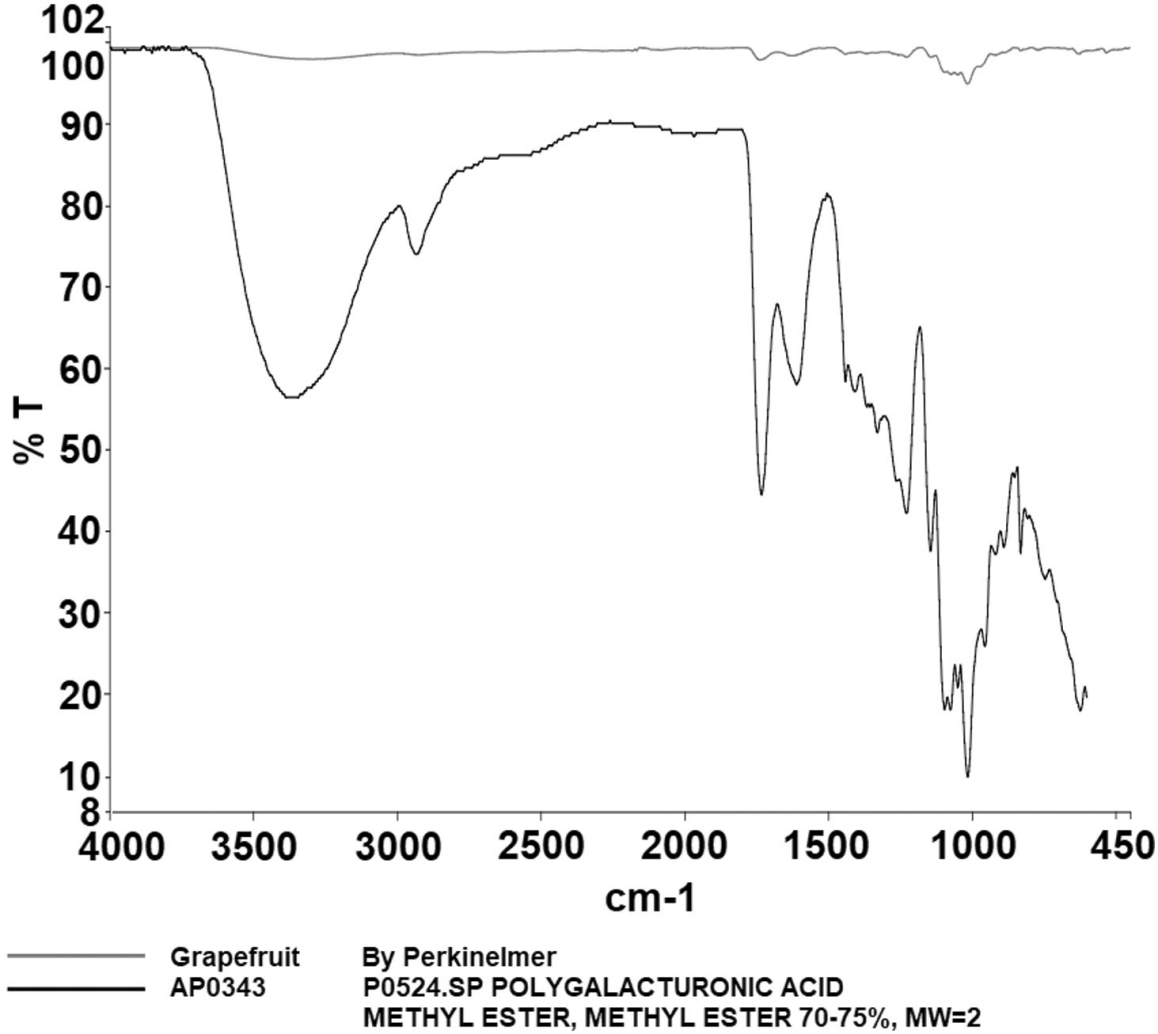
Result of FT‐IR analysis of grapefruit pectin.

**FIGURE 2:**
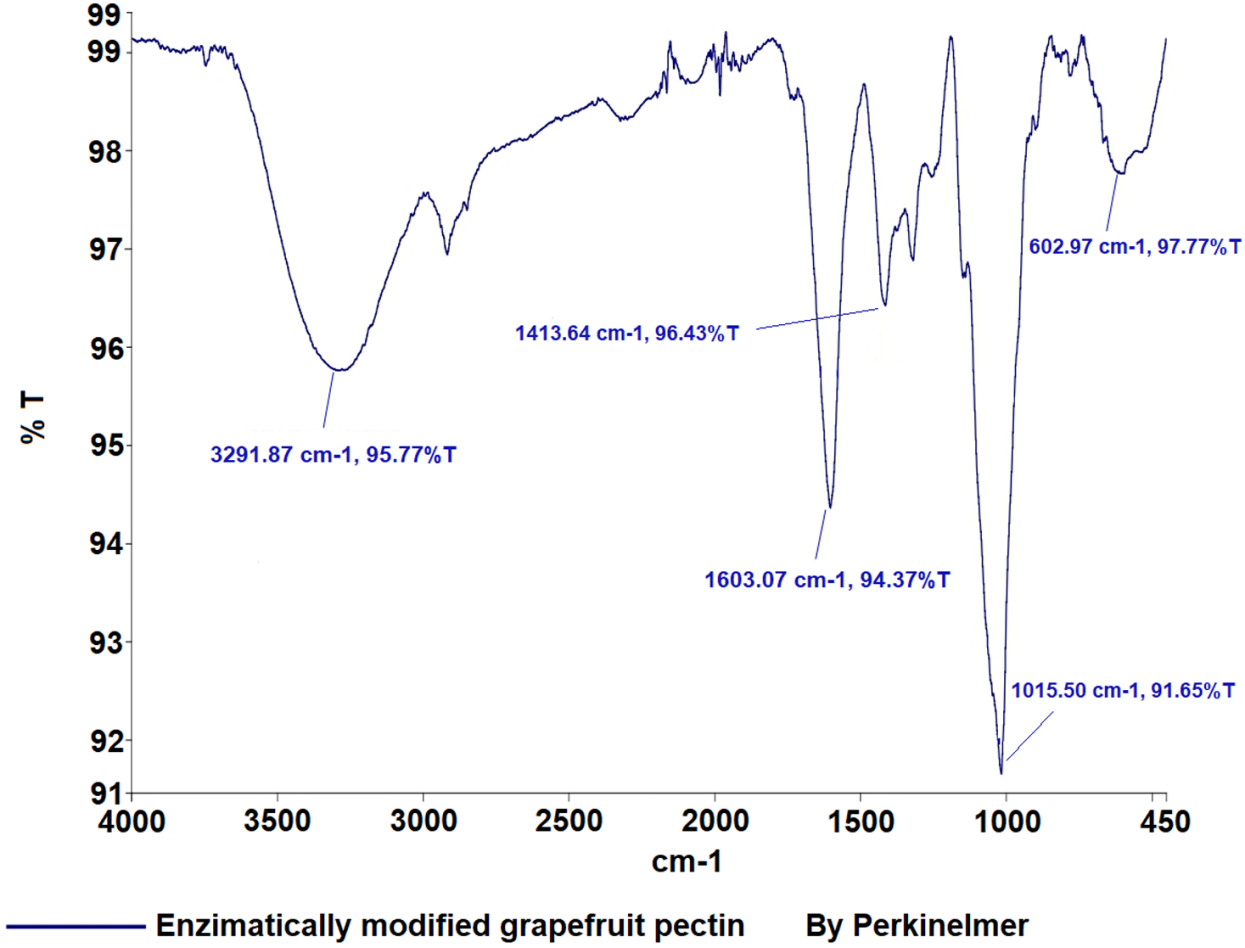
Result of FT‐IR analysis of grapefruit modified pectin.

FTIR spectroscopy is a widely used technique for characterizing the bonding structures of atoms, with pectin's characteristic peaks typically ranging from 400 to 4000 cm^−1^ (Wüstenberg 2014). In Figure 1, the detailed FT‐IR spectra of grapefruit pectin, based on comparison with the device's library, are shown. The spectra of grapefruit pectin produced in this study closely resemble those of commercial pectin and exhibit the characteristic peaks reported in the literature (Ashford et al. 1994; Baekey et al. 1988; Brake and Fennema 1993; El‐Zoghbi and Sitohy 2001; Nangia‐Makker et al. 2002; Videcoq et al. 2011). The FT‐IR spectrum of enzymatically modified grapefruit pectin is displayed in Figure 2, with specific absorption bands observed at 3291 cm^−1^ (v(OH)), 1603 cm^−1^ (v_as(COO^−^)), and 1015 cm^−1^ (v(C‐C) (C‐O)).

**FIGURE 3:**
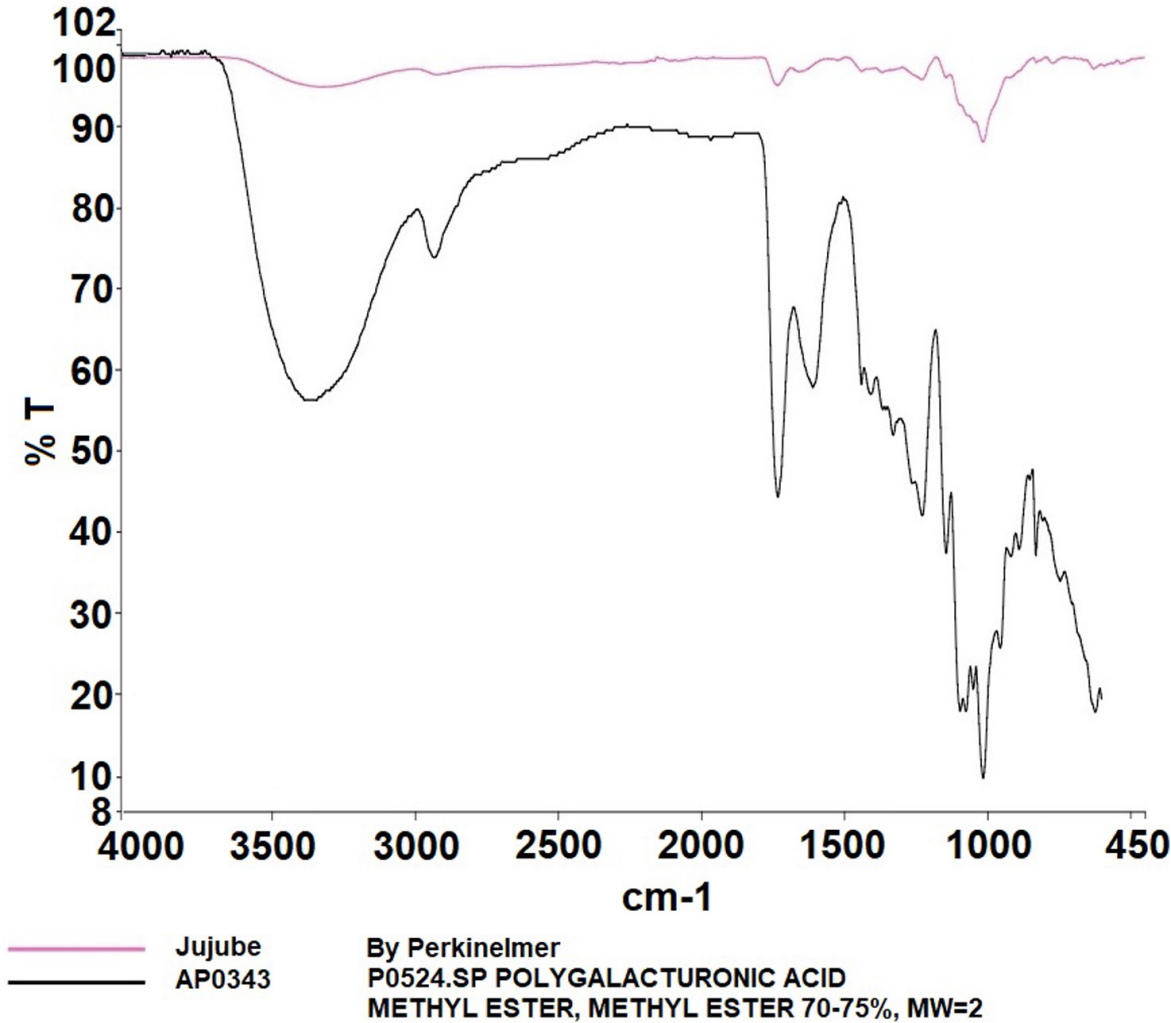
Result of FT‐IR analysis of jujube pectin.

**FIGURE 4:**
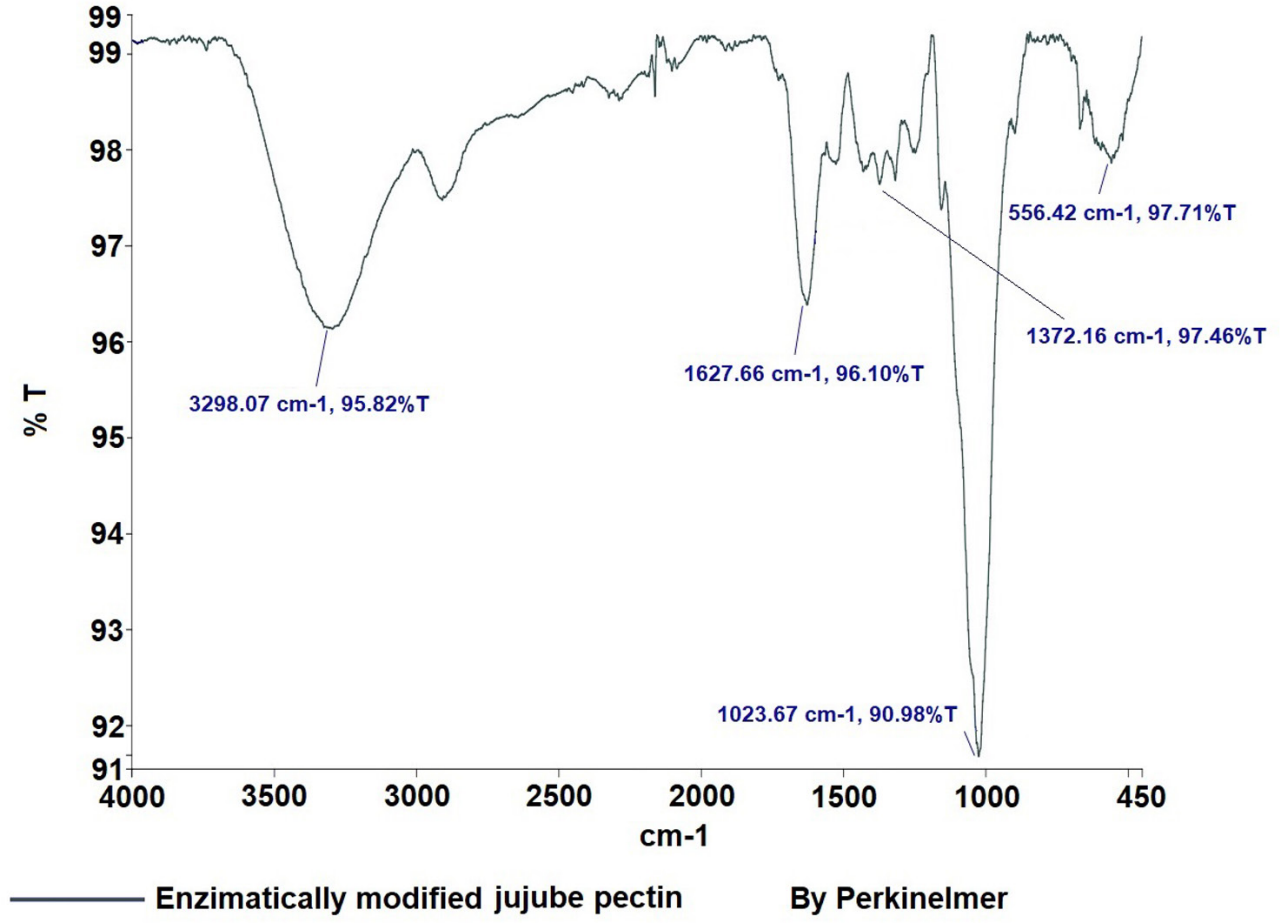
Result of FT‐IR analysis of jujube modified pectin.

In Figure 3, the detailed FT‐IR spectra of jujube pectin are provided, also compared with the library of the device. Similarly, the jujube pectin spectra align with those of commercial pectin and show characteristic peaks consistent with the literature (Ashford et al. 1994; Baekey et al. 1988; Brake and Fennema 1993; El‐Zoghbi and Sitohy 2001; Nangia‐Makker et al. 2002; Videcoq et al. 2011). The enzymatically modified jujube pectin spectrum (Figure 4) displays absorption bands at 3298 cm^−1^ (v(OH)), 1627 cm^−1^ (v_as(COO^−^)), 1372 cm^−1^ (δ(OH)COOH), and 1023 cm^−1^ (v(C‐C) (C‐O)).

**FIGURE 5:**
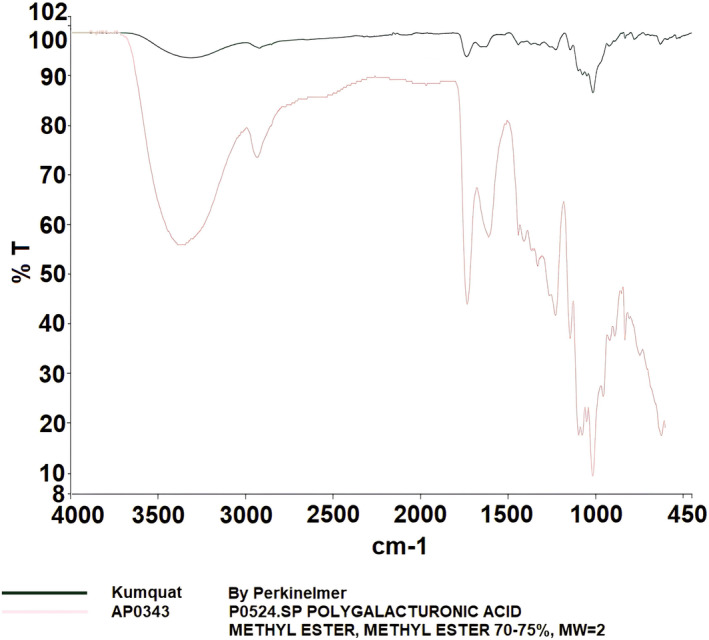
Result of FT‐IR analysis of kumquat pectin.

**FIGURE 6:**
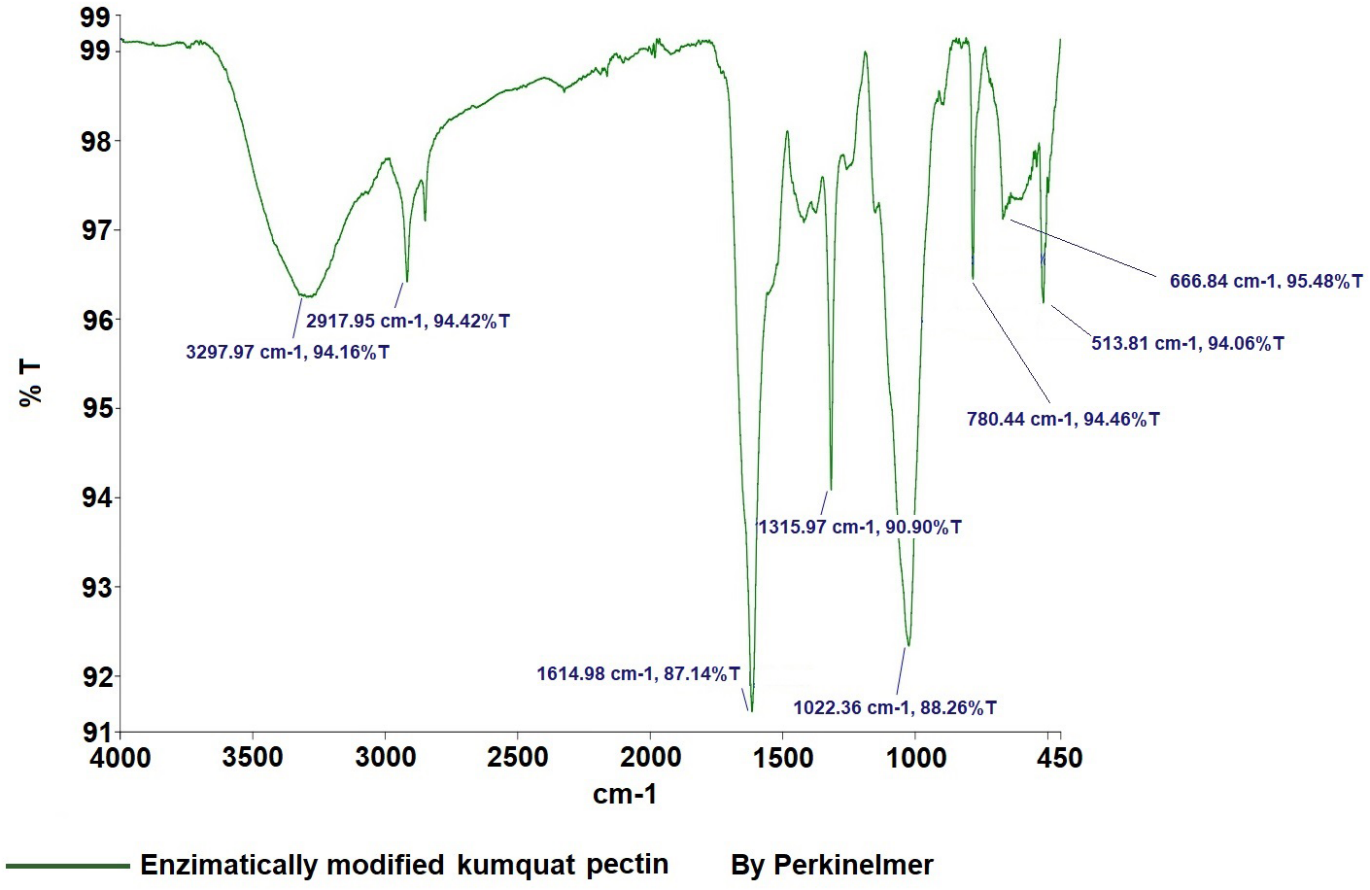
Result of FT‐IR analysis of kumquat modified pectin.

Figure 5 shows the detailed FT‐IR spectra of kumquat pectin, again compared to the library of the device. The kumquat pectin spectra also mirror the spectra of commercial pectin and contain the expected characteristic peaks (Ashford et al. 1994; Baekey et al. 1988; Brake and Fennema 1993; El‐Zoghbi and Sitohy 2001; Nangia‐Makker et al. 2002; Videcoq et al. 2011). The FT‐IR spectrum of enzymatically modified kumquat pectin (Figure 6) reveals absorption bands at 3297 cm^−1^ (v(OH)), 2917 cm^−1^ (v(CH)), 1614 cm^−1^ (v_as(COO^−^)), 1315 cm^−1^ (δ(OH)COOH), and 1022 cm^−1^ (v(C‐C) (C‐O)).

### 3.2 Galacturonic Acid Content of Pectins

The galacturonic acid content is a key indicator of pectin purity (Wu et al. 2022). Various factors, such as fruit structure, ripeness, extraction method, pH, and temperature, influence the galacturonic acid content. In this study, pectin yield from 100 g of dried fruit peel was found to be 20%–25% for grapefruit, 16%–17% for kumquat, and 6%–7% for jujube pectin. The extraction process involved ethanol and was conducted for 5 h at pH 1.88°C and 89°C.

For galacturonic acid quantification, 30 mg of pectin from each sample was pretreated and analyzed using HPLC. A standard calibration curve was drawn by injecting solutions at concentrations of 0.62, 1.25, 2.5, 5.0, and 10.0 mg/L into the device. Based on this calibration curve, the galacturonic acid content was calculated as 612 mg/g for grapefruit pectin, 544 mg/g for jujube pectin, and 704 mg/g for kumquat pectin.

### 3.3 DSC Analysis of Pectins

**FIGURE 7:**
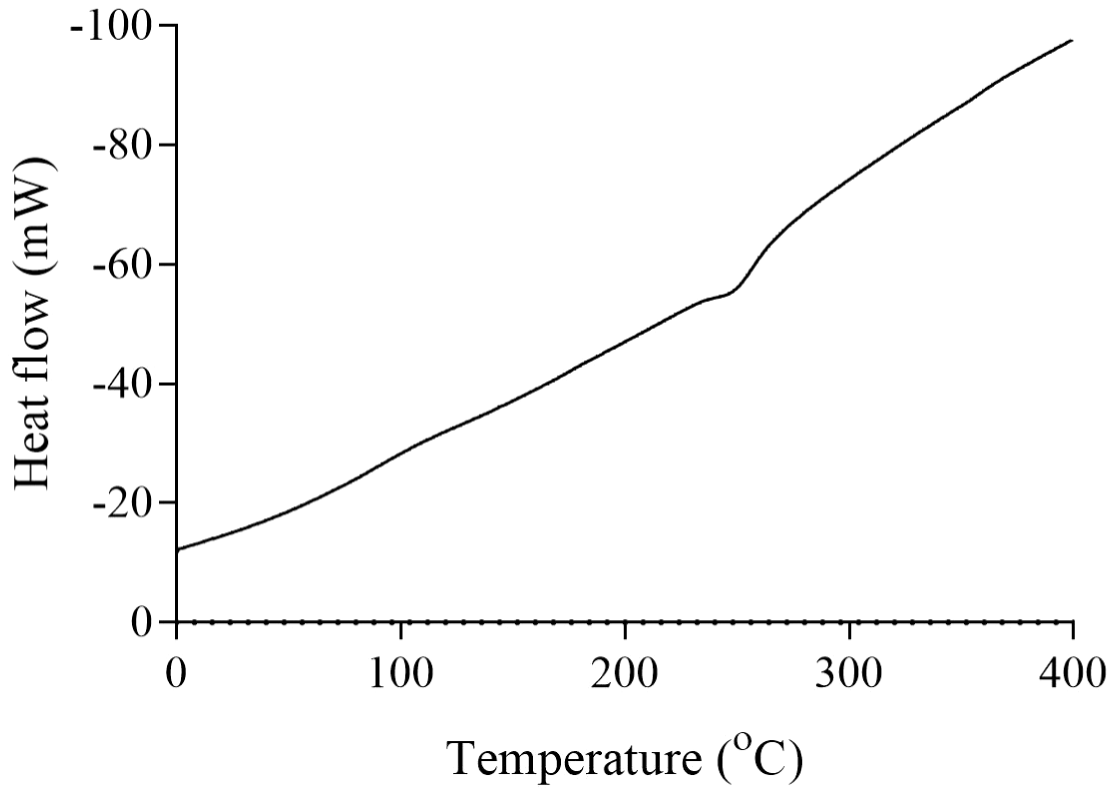
DSC curve of grapefruit pectin.

**FIGURE 8:**
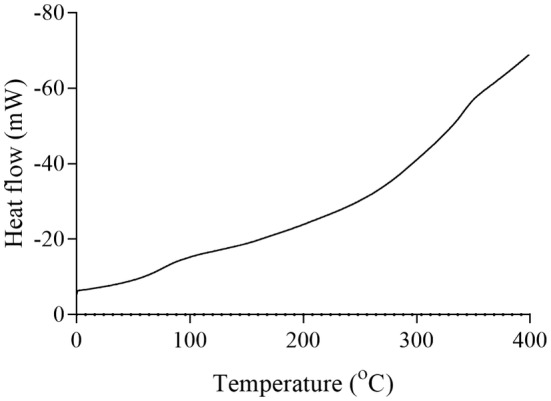
DSC curve of grapefruit modified pectin.

The thermal properties of pectins and their enzymatic modifications were analyzed using differential scanning calorimetry (DSC). For grapefruit pectin, the melting temperature was observed at approximately 110°C, and the degradation temperature at ~270°C (Figure 7). The enzymatically modified grapefruit pectin showed a lower melting point at ~90°C and a degradation temperature at ~350°C (Figure 8).

**FIGURE 9:**
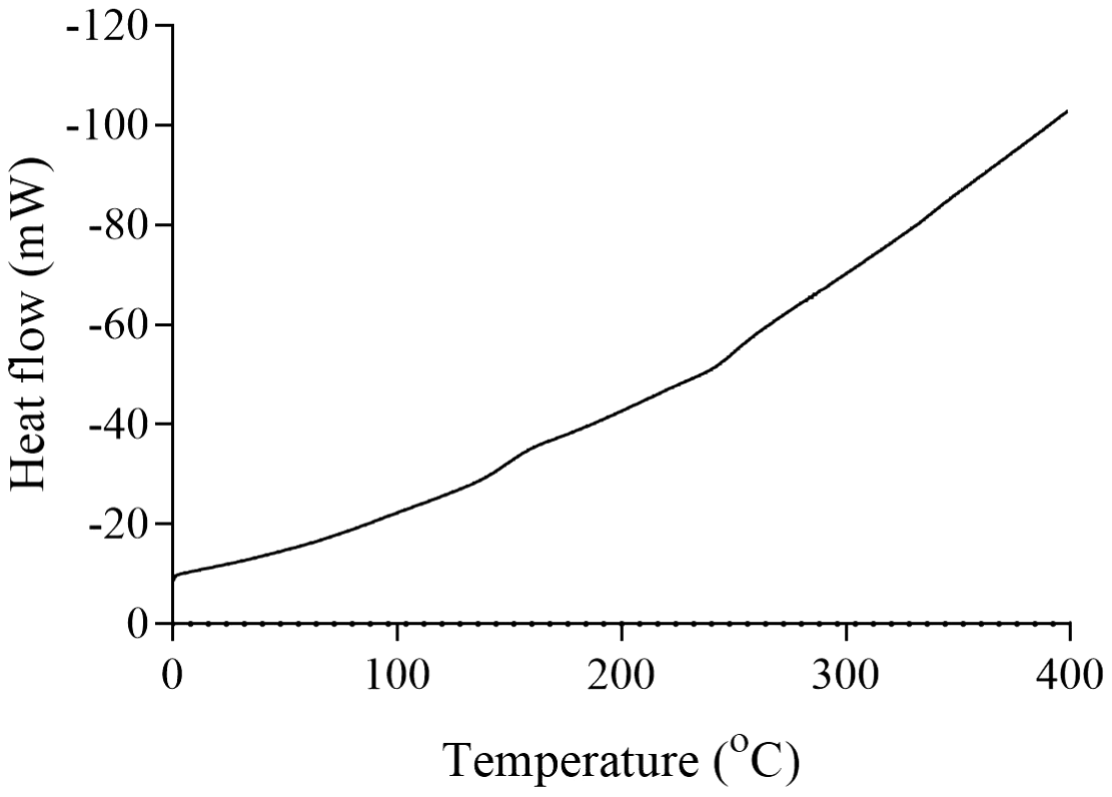
DSC curve of jujube pectin.

**FIGURE 10:**
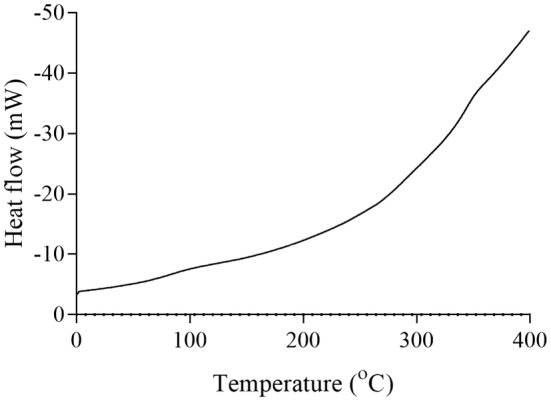
DSC curve of jujube modified pectin.

**FIGURE 11:**
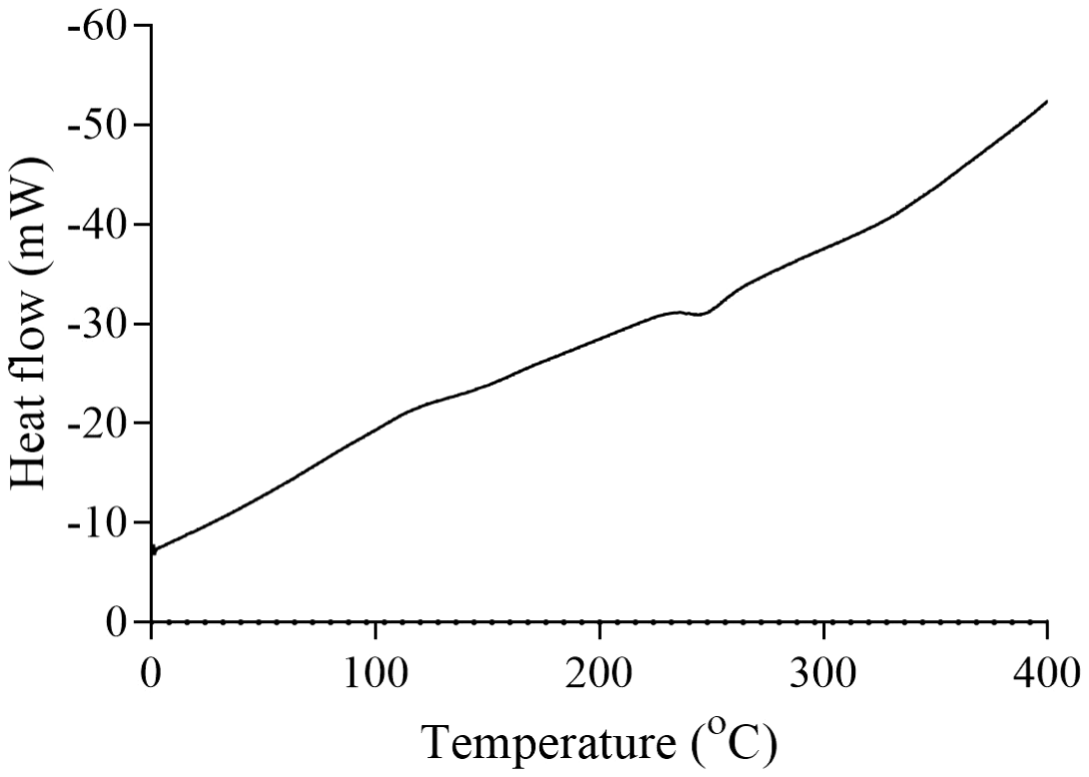
DSC curve of kumquat pectin.

**FIGURE 12:**
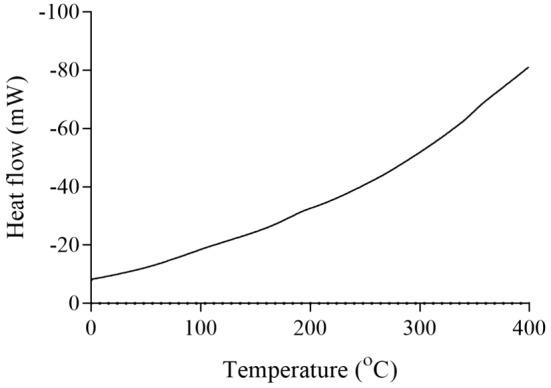
DSC curve of kumquat modified pectin.

For jujube pectin, the melting temperature was ~170°C, with a degradation temperature of ~260°C (Figure 9). The enzymatically modified jujube pectin displayed a melting point of ~100°C and a degradation temperature at ~360°C (Figure 10). Kumquat pectin exhibited a melting temperature of ~120°C and a degradation temperature at ~270°C (Figure 11). The enzymatically modified kumquat pectin had the same melting point (~120°C), while the degradation temperature shifted to ~360°C (Figure 12).

### 3.4 Cytotoxicity of Enzymatically Modified Pectins

This study investigated the cytotoxic effects of enzymatically modified pectins from grapefruit, jujube, and kumquat fruits on the MKN‐45 gastric cancer cell line. The effects of different concentrations (1.2, 0.6, 0.3, 0.15, and 0.075 mg/mL) over 72 h were assessed.

Grapefruit pectin exhibited 61.13 ± 13.38% cell viability at 1.2 mg/mL, with the most effective concentration being 0.075 mg/mL, where viability dropped to 8.36 ± 16.11%. Statistically significant reductions were observed at concentrations of 0.6, 0.3, 0.15, and 0.075 mg/mL (*p* < 0.05). Jujube pectin showed 102.84 ± 7.13% viability at 1.2 mg/mL, with the most effective concentration at 0.075 mg/mL (71.19 ± 3.41%). Statistically significant reductions were also noted at lower concentrations (p < 0.05). For kumquat pectin, viability was 85.76 ± 10.35% at 1.2 mg/mL, increasing as concentrations decreased. The most effective concentration was 0.075 mg/mL, with a viability rate of 63.06 ± 11.1%, with statistical significance (*p* < 0.05) only at this concentration (Table 1).

**TABLE 1:** Cell viability results of enzymatic modified products.

	Grapefruit enzymatically modified product		Jujube enzymatically modified product		Kumquat enzymatically modified product	
	% Viability (mean)	±Standard deviation	% Viability (mean)	±Standard deviation	% Viability (mean)	±Standard deviation
Control	100	3.686	100	3.686	100	3.686
1.2 mg/mL	61.12671	13.38	102.838	7.1337	85.76159	10.35
0.6 mg/mL	56.65146	9.72	85.761	10.353	78.99716	7.69
0.3 mg/mL	54.66329	8.77	79.422	1.4747	66.46168	3.66
0.15 mg/mL	26.07431	7.59	76.9631	5.6902	65.5629	6.73
0.075 mg/mL	8.363837	6.11	71.192	3.4088	63.05582	11.1

*Note:* % Viability: Cell Viability Detection Kit‐8 (CVDK‐8) results, mean: The average of the test results performed with three repetitions, *p* value should be < 0.05.

The increasing demand for ready‐to‐eat foods in Türkiye has driven the need for pectin, a compound already in high demand globally. It is extensively used in the production of confectioneries, jams, jellies, fruit juices, and sauces. Additionally, pectin is employed in chemical, cosmetic, and pharmaceutical industries. Despite its widespread use, pectin is not produced domestically and is entirely imported.

This study extracted pectin from three fruits and characterized it using FT‐IR, HPLC, and DSC. The presence of specific pectin peaks confirmed that the extracts were indeed pectin. Enzymatic modifications were performed, and the changes were analyzed. Based on the results, it is anticipated that pectin could be beneficial in various fields, particularly in the food industry, while modified pectins hold promise for health applications, especially in cancer treatment.

Pectin, a crucial polysaccharide, was successfully extracted and enzymatically modified from three fruits. FT‐IR analysis revealed characteristic peaks, such as those in the 3200–3400 cm^−1^ range (OH vibration bands), as well as the C‐H vibrations in the 2900–2400 cm^−1^ range, corresponding to galacturonic acid esters (Güzel and Akpınar 2019; Tian et al. 2016). Peaks at 1500–1800 cm^−1^ are crucial for determining pectin structure, especially in relation to carboxylic acid groups. The esterified and non‐esterified carboxylic acid groups were identified between 1600 and 1730 cm^−1^, while the 1000–1150 cm^−1^ region was associated with C‐OH and C‐O‐H groups, indicative of C‐C and R‐O‐R ether groups within pectin's ring structure (Singthong et al. 2004; Winning et al. 2009). The FT‐IR analysis demonstrated that the extraction and modification processes were successful.

The galacturonic acid content, the fundamental building block of pectin, was analyzed by HPLC and determined to be 704 mg/g in kumquat pectin, 612 mg/g in grapefruit pectin, and 544 mg/g (dry pectin) in jujube pectin. These values align with the galacturonic acid content reported by Uçan and Akyıldız (2012). Similarly, Buchholt et al. (2004) found the galacturonic acid content of sugar beet pulp to be 548 mg/g of dry pectin, while Yılmaz et al. (2017) reported 406.44 mg/g in powdered pectin from orange pulp, results that are consistent with those found in our study.

The degradation behavior of the pectins and their enzymatically modified forms, as determined by DSC analysis at various temperatures, indicates that pectin exhibits significant thermal stability. Pectin is widely used for its gel‐forming properties, primarily in the food industry. It is commonly used in products such as jams, marmalades, and jellies, which require processing at high temperatures. Therefore, pectin used in these applications must exhibit a high degree of thermal resistance. The pectins and modified products obtained in this study begin to degrade chemically at around 250°C. Based on these properties, they may be suitable for use in food industry processes that involve high heat.

Given Türkiye's favorable geographical location and climatic advantages, progress in domestic pectin production using local fruit species seems feasible, provided that the country's diverse plant resources are effectively utilized. Pectin, a widely used additive, is becoming increasingly in demand due to its versatile applications. Extracting pectin from fruit and vegetable waste is a beneficial practice, especially in light of the growing emphasis on environmental sustainability and waste recycling. The rich biodiversity of Türkiye's soils and the potential use of agricultural waste for pectin production could benefit the national economy.

While the incidence of stomach cancer is declining globally, it remains a significant health concern, particularly as the second most common cancer in Türkiye. Gastric cancer is a multifactorial disease influenced by both genetic and environmental factors. In the MKN‐45 gastric cancer cell line, all concentrations of enzymatically modified pectin derived from grapefruit showed a strong cytotoxic effect at 72 h, with 0.075 mg/mL being the most effective concentration (*p* < 0.05). Similarly, the enzymatically modified kumquat pectin exhibited antiproliferative effects at 72 h, with the most effective concentrations being 0.3, 0.15, and 0.075 mg/mL, all showing statistically significant results (*p* < 0.05). For the jujube pectin, the cytotoxic effect at 72 h was determined to follow the order: 0.075 > 0.15 > 0.3 > 0.6 mg/mL (*p* < 0.05).

Further molecular studies are needed to elucidate the mechanism behind the cytotoxic effects of these enzymatically modified pectins in the MKN‐45 cell line. Specifically, future research should investigate the expression levels of oncogenes and tumor suppressor genes in MKN‐45 cells before and after treatment, as well as explore the apoptotic pathways involved. Given its strong cytotoxic properties, this enzymatically modified pectin may also serve as a potential candidate for drug development and could become a valuable pharmacological product.

## 4 Conclusion

Upon reviewing the scientific literature, it becomes evident that laboratory‐scale pectin production in Türkiye remains limited. This research contributes to laboratory‐scale pectin production and is expected to provide valuable insights for future industrial scale production. If this study is successfully scaled up, it could reduce Türkiye's reliance on imported pectin and increase employment opportunities nationwide. Additionally, this research on utilizing plant residues for pectin production could inspire future scientific studies. High‐value functional products, such as food additives, could be developed from this research, benefitting companies that produce functional foods and improving their existing products and processes. Furthermore, the production of additives from residual materials that contribute to environmental pollution would support sustainability and profitability. Also, based on the results obtained, further in‐depth analyses of the physiological pathways involved in gastric cancer and apoptosis are essential. Given the importance of in vivo studies for assessing the anticancer effects of molecules within living organisms, these analyses should be replicated using experimental animals administered with enzymatically modified pectin molecules.

## Author Contributions

**Selime Üstün:** conceptualization (equal), data curation (equal), formal analysis (equal). **Hamit Emre Kizil:** conceptualization (lead), data curation (lead), formal analysis (lead). **Enes Dertli:** conceptualization (equal), data curation (equal), formal analysis (equal).

## Ethics Statement

This study includes data from a master's thesis conducted at Bayburt University Graduate Education Institute, and all data were obtained before the year 2020.

## Conflicts of Interest

Conceptualization, Data curation, Formal analysis was performed by Hamit Emre KIZIL, Selime ÜSTÜN and Enes DERTLİ. Funding acquisition: The work was financially supported by Bayburt University Scientific Research Projects Unit with Project Code: 2018/02‐69001‐07. Resources: MKN‐45 cell line was obtained from Yeditepe University, Department of Genetics and Bioengineering laboratory and stored by Hamit Emre KIZIL and Selime ÜSTÜN. This study includes some of the data of the master's thesis of Selime Üstün, a master's student who graduated from Bayburt University Institute of Educational Sciences in 2021. Selime Üstün's master's thesis advisor is Hamit Emre KIZIL and her co‐advisor is Enes DERTLİ (YÖK ID: 698052).

## Data Availability

Data are available within the manuscript.
